# Development and Characterization a Single-Active-Chamber Piezoelectric Membrane Pump with Multiple Passive Check Valves

**DOI:** 10.3390/s16122108

**Published:** 2016-12-12

**Authors:** Ronghui Zhang, Feng You, Zhihan Lv, Zhaocheng He, Haiwei Wang, Ling Huang

**Affiliations:** 1 Research Center of Intelligent Transportation System, School of Engineering, Sun Yat-sen University, Guangzhou 510275, China; zrh1981819@126.com (R.Z.); hezhch@mail.sysu.edu.cn (Z.H.); 2 School of Civil Engineering and Transportation, South China University of Technology, Guangzhou 510640, China; youfeng77@126.com (F.Y.); hling@scut.edu.cn (L.H.); 3 Department of Computer Science, University College London, 66-72 Gower Street, London WC1E 6EA, UK; Z.Lu@cs.ucl.ac.uk

**Keywords:** piezoelectric membrane pump, PZT actuator, multiple check valves, flow rate, backpressure

## Abstract

In order to prevent the backward flow of piezoelectric pumps, this paper presents a single-active-chamber piezoelectric membrane pump with multiple passive check valves. Under the condition of a fixed total number of passive check valves, by means of changing the inlet valves and outlet valves’ configuration, the pumping characteristics in terms of flow rate and backpressure are experimentally investigated. Like the maximum flow rate and backpressure, the testing results show that the optimal frequencies are significantly affected by changes in the number inlet valves and outlet valves. The variation ratios of the maximum flow rate and the maximum backpressure are up to 66% and less than 20%, respectively. Furthermore, the piezoelectric pump generally demonstrates very similar flow rate and backpressure characteristics when the number of inlet valves in one kind of configuration is the same as that of outlet valves in another configuration. The comparison indicates that the backflow from the pumping chamber to inlet is basically the same as the backflow from the outlet to the pumping chamber. No matter whether the number of inlet valves or the number of outlet valves is increased, the backflow can be effectively reduced. In addition, the backpressure fluctuation can be significantly suppressed with an increase of either inlet valves or outlet valves. It also means that the pump can prevent the backflow more effectively at the cost of power consumption. The pump is very suitable for conditions where more accurate flow rates are needed and wear and fatigue of check valves often occur.

## 1. Introduction

Micropumps are attracting interest in the research community because of their major applications in pharmaceutical and bio-medical drug delivery systems, microelectronics cooling, and their use in chemical and biological substance analysis systems as microfluidic flow control appliances [1,2,3,4]. The first micropumps were presented in the 1970s and 1980s [5,6,7,8]. In the past three decades, researchers have been designing and developing various micropumps using different actuation methods such as piezoelectric, electromagnetic, electrostatic, thermopneumatic and shape memory alloy (SMA) devices [1,2,3,4]. To date, micropumps have already been implemented in the market and are widely used in ink jet printers and fuel injection applications [1,4]. Although most micropumps have complex structures and a high level of power consumption, piezoelectric actuation has the advantages of a relatively simple structure, short response time and relatively low power consumption [3,9,10]. Typically, piezoelectrically-actuated pumps use a piezoelectric actuator to move a membrane in a chamber providing fluid delivery with the flow direction being controlled by check valves [11,12]. Applications can be found from micro-dosing drug administration to propelling microspacecraft [13,14,15,16,17].

Presently, piezoelectric pumps can roughly be categorized by their valve types as with check valve or without valve [18,19,20]. Piezoelectric check-valve pumps are usually comprised of a periodically working membrane and two passive check valves. Valveless micro-pumps make use of diffuse/nozzle structures to replace passive check valves as the flow rectifying elements. Because check-valve piezoelectric pumps can overcome higher flow resistance and be used for both liquids and gases [21,22], check-valve piezoelectric membrane pumps have been the focus of a lot of research and development activities [23,24,25,26]. Various piezoelectric actuators like unimorph, bimorph and stack variants were used to drive the membrane pumps [27,28,29,30]. More working media such as water, air, oil, alcohol, ethanol and methanol could be delivered. Not only traditional fabrication techniques and materials, but also MEMS technologies and new materials such as PDMS, silicon rubber, plastic and parylene are widely applied in this area. Furthermore, much attention has been paid to the development of passive check valves. A variety of ball-, membrane-, cantilever-, umbrella-, bridge-, and wheel-type passive check valves were presented [1,21,28,31]. Nevertheless, even though great progress has been made during the last thirty years, there is a lot of room for further improving the performance of existing check-valve piezoelectric membrane pumps. There are still some limitations for practical applications, especially the backward leakage of check valves. In fact, not only check-valve piezoelectric pumps suffer from the backflow problem, but this problem is also more serious for valveless piezoelectric pumps because of the lack of blocks. A number of works on pumps and valves were conducted to reduce the backflow and improve the pumping performance [1,22,24,27,32,33,34,35].

In this paper, we propose a single-active-chamber piezoelectric membrane pump with multiple passive check valves, which is intended to reduce the backflow by means of increasing the number of passive check valves. Because the single-active-chamber piezoelectric pump has more than one check valve, it is very important to configure the multiple check valves and find out the influence of different configurations of multiple check valves on the pump properties. Through basic experiments on frequency characteristics, the pumping rate and output backpressure of a check-valve piezoelectric membrane pump is investigated by changing the number of inlet valves and outlet valves.

## 2. Working Principle

**Figure 1:**
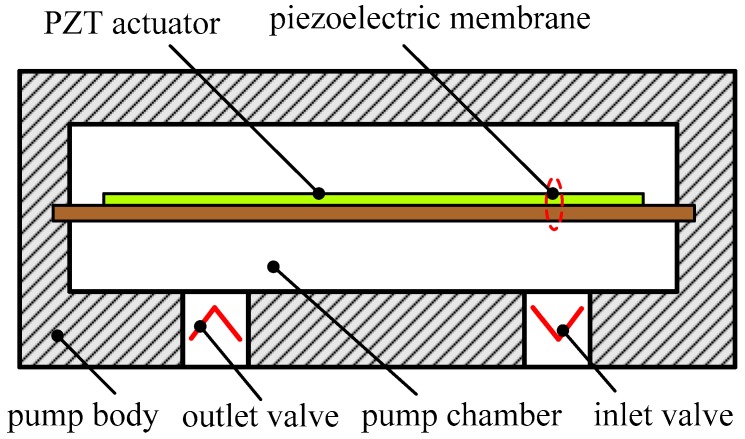
The structure of a classic piezoelectric membrane pump.

A piezoelectric pump uses a PZT actuator to move a membrane in a chamber providing fluid entrance and exit with the flow rectification being controlled by check valves. A classic piezoelectric membrane pump is comprised of a piezoelectric membrane, a pumping chamber connected to inlet and outlet valves, and pump body, as illustrated in Figure 1. The working principle can be described as a cyclic process of an expansion stroke and a compression stroke. When an AC voltage is applied to the PZT actuator, the membrane deflection will alternately result in the pumping chamber expansion and compression. During the expansion stroke, with the increase of the pumping chamber volume, under-pressure is generated in the pumping chamber and the inlet valve opens with the corresponding decrease in chamber pressure. Consequently, working medium is sucked into the expanding chamber. During the compression stroke, over-pressure is generated in the pumping chamber because of the chamber volume decrease and thus the outlet valve opens. Working media are discharged through the outlet valve. 

**Figure 2:**
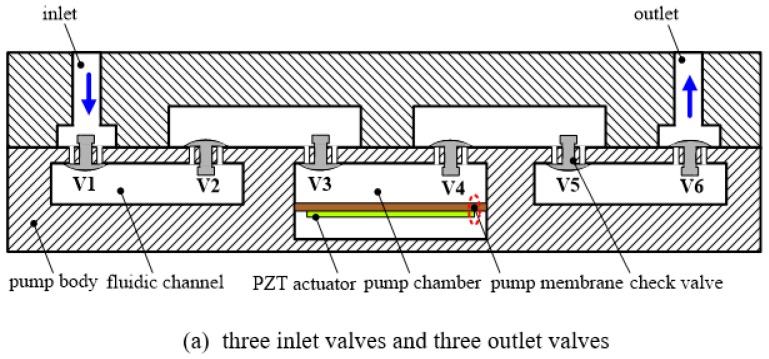

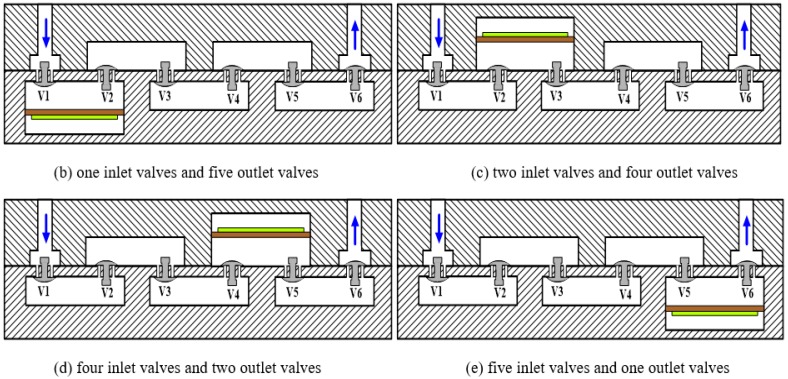
The demonstration structure of a single-active-chamber piezoelectric membrane pump with multiple passive check valves.

When the PZT actuator is driven by alternating voltage, a piezoelectric membrane pump can transfer the mechanical energy of membrane deflection into fluid movement. Then the mechanical vibration of the piezoelectric membrane is transmitted to the check valves through the working fluid. Apparently, it takes some time to perform the transmission process. The valve is constrained by the force of inertia caused by the working fluid. Therefore, the actions (open/close) of the passive check valves always lag behind the vibration of the PZT actuator. Ideally, the outlet valve should be closed when the inlet valve opens during the expansion stroke. However, because of the outlet valves’ closing lag, some fluids which have been discharged are redrawn into the pump chamber through the outlet valve, i.e., backflow occurs. Similarly, because of the closing lag from the inlet valves during the compression stroke, some fluids which have been sucked are forced out of the pump chamber through the inlet valve over again. So far, various research efforts have mostly focused on adopting new materials and techniques, developing new valve types and optimizing the existing check valves. Inspired by the works in [36,37], where Hwang et al. designed a leakage barrier in the chamber to reduce leakage flow without interfering with the net positive fluid flow, and Ma et al. added a secondary chamber to obstruct the stream and reduce the backflow toward the primary chamber, a single-active-chamber piezoelectric membrane pump with multiple passive check valves is proposed in this paper, as shown in Figure 2. This configuration of multiple passive check valves is intended to prevent the backflow toward the working chamber. Then each check valve can be regarded as a leakage barrier. During the expansion stroke, multiple outlet valves are able to reduce the backflow of the fluids which have been discharged. Likewise, multiple inlet valves can effectively prevent the fluids which have been sucked into the pump chamber from flowing back into the inlet.

## 3. Fabrication

**Figure 3:**
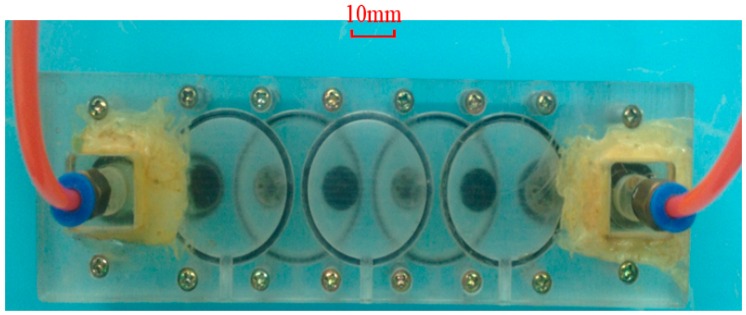
The prototype photograph of the single-active-chamber piezoelectric membrane pump with multiple passive check valves.

In this work, six passive check valves are used to construct the piezoelectric membrane pump. As shown in Figure 2, V*_i_* (*i* = 1, 2, …, 6) represent the passive check valves. The casting-rubber moulding method was adopted to form six umbrella-shaped check valves and six little valve orifices with a diameter of Φ1.2 mm were designed to enhance reverse-check capability. The piezoelectric membrane with a size of Φ35 mm × 0.53 mm is a commercial piezoelectric sound component produced by Murata Manufacturing Co., Ltd. (Nagaoka, Japan), where the thicknesses of the piezoelectric component with the diameter of Φ25 mm and the metal plate with the diameter of Φ35 mm are 0.23 mm and 0.3 mm, respectively. In fact, we adopted five similar piezoelectric membranes to characterize the piezoelectric membrane pump with different numbers of inlet and outlet valves. The pump shell and body are made of polymethyl methacrylate which has the advantages of easy processing, light weight, insulation and easy observation. After two umbrella-shaped check valves close to the piezoelectric membrane as well as the membrane are assembled, a pumping chamber is ready. The room between other two neighbouring check valves forms several fluidic channels. The prototype photograph of the single-active-chamber piezoelectric membrane pump with multiple passive check valves is shown in Figure 3.

## 4. Experimental Setup

**Figure 4:**
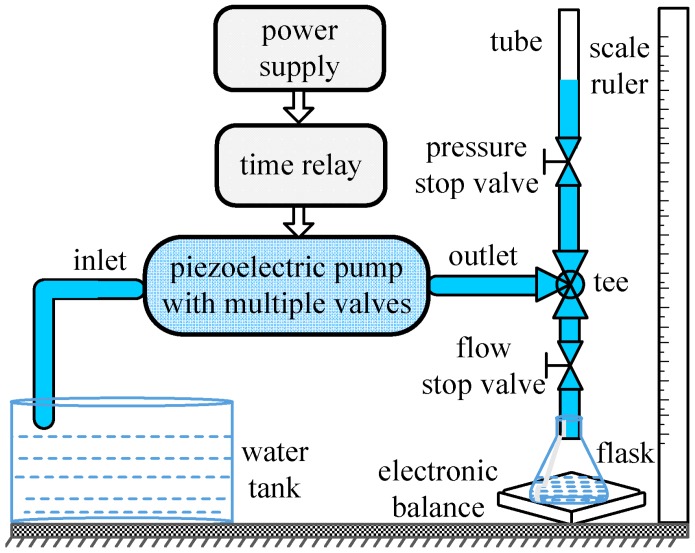
Experimental setup.

The performance of the proposed piezoelectric pump needed to be experimentally measured in terms of flow rate and backpressure that the prototype pump could achieve. Figure 4 depicts the schematic diagram of the experimental setup. The tap water at room temperature, as the most representative working fluid, is used as working fluid. Two plastic tubes are connected to the inlet and outlet of the prototype pump. A digital PZT power supply (P211, SANKI, Nagoya, Japan) is used to drive the piezoelectric membrane pump. The power supply can provide a sinusoidal voltage with a variable frequency of 60–400 Hz and an AC voltage range of 0–240 V. An electronic balance with a division value of 0.01 g and a repeatability of 0.01 g is used to measure the outflow. A time relay is used to set the time interval to obtain the average flow rate. The time interval of sixty seconds is set in each measurement. According to the energy equation, the water column height which is perpendicular to the water free surface is measured by a scale ruler in order to obtain the pumping pressure value (kPa). The measurement of flow rate or backpressure is carried out through opening and closing corresponding stop valves. The tee, pressure stop valve and flow stop valve are used to choose the measured object. When the pressure stop valve is closed, the flow characteristics can be tested. On the contrary, backpressure characteristics can be obtained. Using this setup, a series of experiments are conducted to explore the performance of the single-active-chamber piezoelectric membrane pump with multiple passive check valves.

## 5. Results and Discussion

In this work, six similar passive check valves were adopted to conduct because the piezoelectric pump cannot properly function if the number of check valves is too many. Actually, a piezoelectric membrane pump can have a different number of inlet and outlet valves. The number and configuration of check valves must have a different influence on the piezoelectric membrane pump with multiple valves. Therefore, we mainly focus on the influence of multiple check valves’ various configuration on the performance of the piezoelectric membrane pump in this paper. As mentioned before, in order to characterize and fully compare the different performances of the piezoelectric pump with multiple check valves, five piezoelectric membranes are used. Then the configuration of inlet and outlet valves can be changed by means of activating any one PZT actuator, meanwhile, the inactive piezoelectric membranes consists of fluidic channels. Thereby, we realize the configuration changes without altering any of the mechanical structure, ensuring the same measurement conditions.

### 5.1. The Results of the Pump with One Inlet Valve and Five Outlet Valves

**Figure 5:**
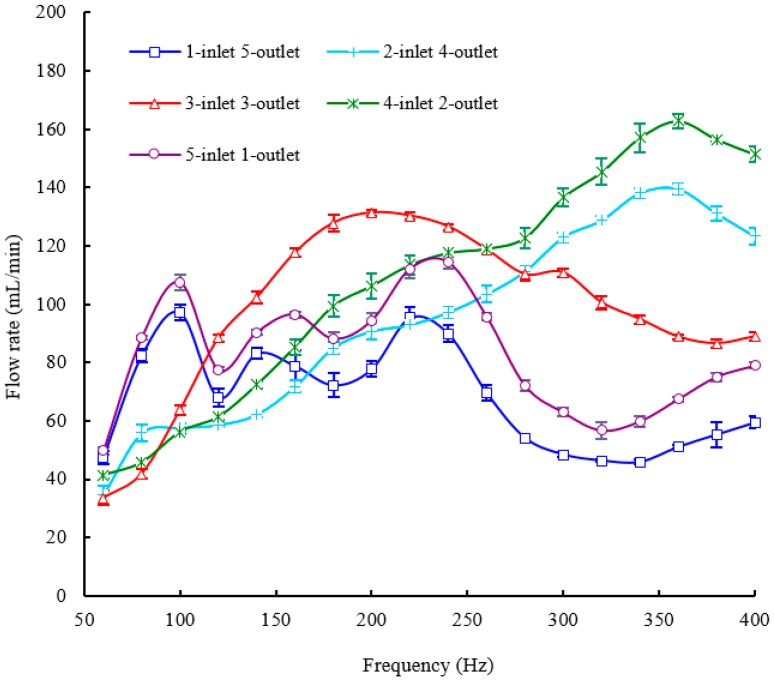
The flow rate versus frequency characteristics of the pump with different configurations of inlet valves and outlet valves.

**Figure 6:**
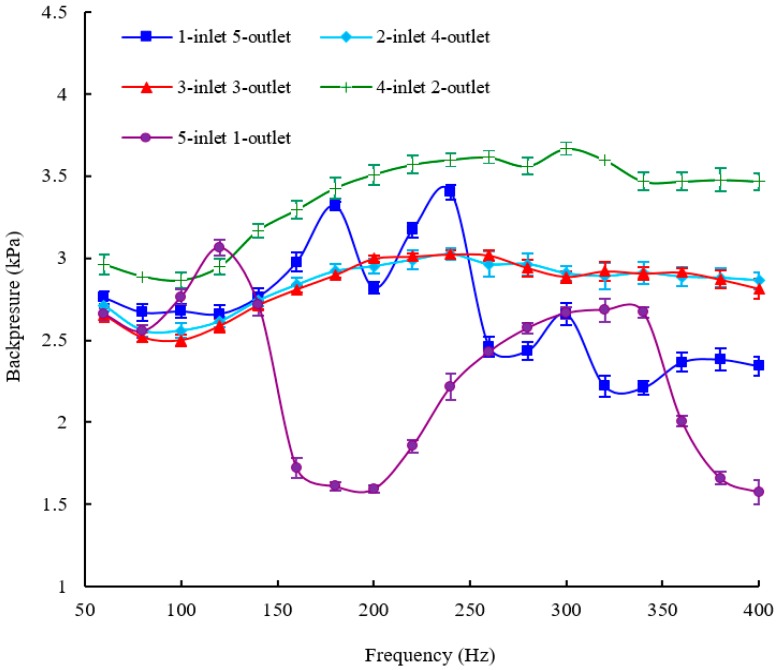
The backpressure versus frequency characteristics of the pump with different configurations of inlet valves and outlet valves.

The structure of the piezoelectric membrane pump with one inlet valve and five outlet valves is shown in Figure 2b. We connected the PZT actuator demonstrated in Figure 2b with the power supply to actuate the pump. Through adjusting the actuation frequency step by step, the flow rate (mL/min) versus frequency (Hz) characteristics is tested at a fixed driving voltage of 150 V. During the measurement of the flow rate, the backpressure difference between the inlet and the outlet is maintained to be zero, namely the flow rate against zero pressure. Figure 5 shows experimental results of the pump performance in terms of flow rate versus actuation frequency, keeping the applied voltage fixed at 150 V. The flow rate is changed with the change of the frequency characteristics. There are two peaks of flow rates at 100 Hz and 220 Hz within the whole frequency range from 60 Hz to 400 Hz and the peak flow rates are almost the same. The maximum flow rate of 100 mL/min is achieved at the frequency of 220 Hz and a driving voltage of 150 V. The minimum flow rate occurs at the frequency of 340 Hz and the valley value of the flow rate is 46.4 mL/min. Apart from the flow rate, backpressure is another important parameter representing the pump performance. Next, we will perform the testing of the frequency-dependent backpressure in order to further examine the piezoelectric membrane pump with multiple check valves. Figure 6 shows the relationship between backpressure as a function of operation frequency at a fixed actuation voltage of 150 V. A relatively big backpressure fluctuation is observed in Figure 6. Four backpressure peaks, two high peaks and two low peaks, are observed from the backpressure versus within the frequency range of 60–400 Hz. The maximum backpressure of 3.45 kPa is achieved at 240 Hz and the minimum backpressure of 2.26 kPa is achieved at 340 Hz.

### 5.2. The Results of the Pump with Two Inlet Valves and Four Outlet Valves

Figure 2c demonstrates the structure of the piezoelectric membrane pump with two inlet valves and four outlet valves. In order to evaluate this structure, the flow rate and backpressure versus frequency characteristics of the pump are tested at a fixed driving voltage of 150 V. In this section and the next three sections, the experimental procedures are totally the same as those of the pump with one inlet valve and five outlet valves. Figure 5 and Figure 6 show the frequency-dependent flow rate and backpressure curves, respectively. For the frequency-dependent flow rates, only one peak of the flow rate is observed at 360 Hz for an applied voltage of 150 V and a maximum flow rate of 140.8 mL/min at 360 Hz is recorded. Before the frequency of 360 Hz, the flow rate of the pump is almost monotonically increased with increasing the frequency. The valley of flow rate is recorded at 60 Hz and the minimum flow rate of 38 mL/min is measured. As for the frequency-dependent backpressures, the backpressure fluctuation is much smaller than that of the pump with one inlet valve and five outlet valves, especially beyond the frequency of 160 Hz. Figure 6 illustrates that only one peak backpressure occurs. The maximum backpressure of 3.06 kPa and the minimum backpressure of 2.6 kPa are recorded at 240 Hz and 100 Hz, respectively. 

### 5.3. The Results of the Pump with Three Inlet Valves and Three Outlet Valves

The structure of the piezoelectric membrane pump with three inlet valves and three outlet valves is shown in Figure 2a. The experimental results of the pump are directly presented because the experimental procedures are very similar to last experiments. Figure 5 and Figure 6 show the frequency-dependent flow rate and backpressure curves, respectively. As observed in Figure 5, the flow rate initially increases with the excitation frequency. After the optimal frequency, the flow rate tends to decrease. The resonant phenomenon occurs in the pump chamber because of the working fluid vibration with the pump components (six check valves and one diaphragm). Within the whole frequency range from 60 to 400 Hz, the pump with three inlet valves and three outlet valves has only one optimal frequency of 200 Hz where the excitation frequency reaches the pump resonance frequency. It is similar to the frequency characteristics of the displacement amplification system. The resonance frequency determines where the maximum flow rate locates. Under resonance, the flow rate reaches the highest value of 132.4 mL/min at the excitation voltages of 150 V. The minimum flow rate of 36 mL/min occurs at 60 Hz. For the frequency-dependent backpressure characteristics, it is seen from Figure 6 that there is also basically a similar trend between this pump and the pump with two inlet valves and four outlet valves. Nevertheless, the maximum backpressure as well as the minimum backpressure is higher than those of the pump with two inlet valves and four outlet valves correspondingly. They are 3.65 kPa at 240 Hz and 2.87 kPa at 100 Hz, respectively.

### 5.4. The Results of the Pump with Four Inlet Valves and Two Outlet Valves

The structure of the piezoelectric membrane pump with four inlet valves and two outlet valves is shown in Figure 2d. Figure 5 and Figure 6 show the frequency-dependent flow rate and backpressure curves, respectively. Experimental results show that the flow rate versus frequency curve of the pump with three inlet valves and three outlet valves takes on a similar trend with the pump with two inlet valves and four outlet valves. The flow rate of the pump is also almost monotonically increased with increasing the frequency until 360 Hz where one peak of the flow rate is measured. Furthermore, the minimum flow rate of the pump also occurs at 60 Hz. However, the maximum flow rate as well as the minimum flow rate is greater than those of the pump with two inlet valves and four outlet valves correspondingly. They are 165.2 mL/min and 42.4 mL/min with an applied voltage of 150 V, respectively. For the frequency-dependent backpressure characteristics, it is observed that the backpressure versus frequency curve of the pump is quite similar to that of the pump with two inlet valves and four outlet valves. The maximum backpressure and the minimum backpressure also occur at 240 Hz and 100 Hz, respectively. Moreover, the maximum and minimum values of output backpressures are 3.05 kPa and 2.54 kPa, respectively, where they are close to the corresponding values of the pump with two inlet valves and four outlet valves. In other words, the backpressure characteristic of the pump with four inlet valves and two outlet valves is basically the same as that of the pump with two inlet valves and four outlet valves.

### 5.5. The Results of the Pump with Five Inlet Valves and One Outlet Valve

The structure of the piezoelectric membrane pump with five inlet valves and one outlet valve is illustrated in Figure 2e. Figure 5 and Figure 6 show the frequency-dependent flow rate and backpressure curves, respectively. The flow rate versus frequency curve in Figure 5 shows a very similar change trend with the pump with one inlet valve and five outlet valves. Two peaks of flow rates are measured at 100 Hz and 240 Hz, respectively. The maximum flow rate occurs at 240 Hz and the pump achieves the highest value of 116.8 mL/min at the excitation voltages of 150 V. The minimum flow rate of 50.8 mL/min occurs at 60 Hz, which is almost consistent with the pump with one inlet valve and five outlet valves. As for the frequency-dependent backpressures, there is a fairly big backpressure fluctuation within the whole frequency range from 60 Hz to 400 Hz. Two peaks of the backpressures are observed at 120 Hz and 320 Hz and a maximum backpressure of 3.1 kPa at 120 Hz is measured with a driving voltage of 150 V. In addition, a minimum backpressure of 1.6 kPa is achieved at 180 Hz. Meanwhile, the pump also achieves a backpressure which is close to the minimum value at 400 Hz.

## 6. Discussion

It is found from the experimental results that different configurations of multiple check valves have a strong influence on the flow rate and backpressure of the piezoelectric membrane pump. In order to demonstrate the performance differences caused by different numbers of inlet and outlet valves intuitively, all the aforementioned frequency-dependent flow rate curves are plotted and compared in one figure, and so are the backpressure curves. They are illustrated in Figure 5 and Figure 6, respectively.

The comparison results show that the output flow rate of the pump changes with different numbers of inlet and outlet valves. Not only the optimal frequency but also the maximum flow rate are significantly affected by the number change of inlet valves and outlet valves. The reason is very complicated. The pump must work on the condition of consistent movement between the membrane and the check valve. Especially for multiple check valves, because there are different time lags between membrane and valve movement, this will lead to different resonant behaviors for the pump with multiple check valves, Among the five tested structures, the variation of the optimal frequency ranges from 200 Hz to 360 Hz, meanwhile, the variation of the maximum flow rate ranges from 100 mL/min to 165.2 mL/min. The variation ratio of the maximum flow rate is up to 66%. However, it is observed from the curve that the pump displays very similar flow rate characteristics between the pump with one inlet valve & five outlet valves and the pump with five inlet valves & one outlet valve. In addition, the flow rate characteristics of the pump are similar when the pump has two inlet valves & four outlet valves as well as four inlet valves and two outlet valves. The flow rate property of the pump with three inlet valves and three outlet valves is totally different from other four types of structures. Nevertheless, there is a very similar backpressure property between the pump with three inlet valves & three outlet valves, two inlet valves and four outlet valves and four inlet valves and two outlet valves, especially the latter two. The variation of the maximum backpressure ranges from 3.05 kPa to 3.65 kPa in all the tested structures. The variation ratio of the maximum backpressure is less than 20%. It can be inferred that the different configurations influence of inlet valves and outlet valves on the flow rate is much greater than the influence on the backpressure.

No matter whether a check-valve piezoelectric pump or a valveless pump is considered, back flow is inevitable. The backflow characteristics of a check-valve piezoelectric membrane pump are investigated by changing the number of inlet and outlet valves. As described above, when the number of inlet valves in one kind of configuration is the same as that of outlet valves in another configuration, the pump generally shows very similar flow rate and backpressure characteristics. Consequently, we can infer that the backflow from pumping chamber to inlet is basically the same as the backflow from outlet to pumping chamber. It means that either increasing the number of inlet valves or increasing the number of outlet valves can effectively reduce the backflow. However, the frequency characteristics of the pump are changed with the different numbers of inlet valves and outlet valves. In addition, the increase of inlet valves or outlet valves can significantly suppress the backpressure fluctuation. It is found that there is a relatively big backpressure fluctuation when just one inlet valve or outlet valve is involved. On the contrary, the backpressure fluctuation is relatively small when more than two inlet valves or outlet valves are involved. For example, the pump with one inlet valve and five outlet valves has a backpressure fluctuation of 1.19 kPa; and the backpressure fluctuation of the pump with two inlet valve and four outlet valves is only 0.46 kPa. It is helpful to improving the stability of the pump, so the pump can retain the backpressure stability across a wider frequency range. However, the increasing number of passive valves and buffer chambers will increase the power consumption. The pump with multiple check valves has a higher power consumption compared with the pump with single inlet valve and outlet valve. In other words, the pump can prevent the backflow more effectively at the cost of power consumption.

## 7. Conclusions

At present, research on check valves of piezoelectric pumps is mostly focused on valve design, development and optimization. Unlike previous research, we mainly explore the effect of check-valve number on the properties of a piezoelectric membrane pump, especially the backflow characteristics of the pump. Because each check valve is not only used as a flow-rectifying element but also regarded as a leakage barrier, multiple check valves could effectively reduce the backward flow.

In this paper, a single-active-chamber piezoelectric membrane pump with multiple passive check valves is proposed. The influence on the pumping performance is experimentally studied by altering the number of inlet valves and outlet valves, keeping the total number of check valves constant. A piezoelectric membrane pump with six passive check valves is fabricated and the prototype pump is experimentally tested in terms of flow rate and backpressure. Experimental results indicate that different configurations of multiple check valves have a strong influence on the output capabilities of the piezoelectric membrane pump. Not only the optimal frequency but also the maximum flow rate is significantly affected by the number change of inlet valves and outlet valves. Among the five tested structures, the variations of the optimal frequency and the maximum flow rate range from 200 Hz to 360 Hz and from 100 mL/min to 165.2 mL/min, respectively. The variation of the maximum backpressure ranges from 3.05 kPa to 3.65 kPa in all the tested structures. Although the flow rate and backpressure are changed with different configurations of inlet valves and outlet valves, it is found that the piezoelectric pump generally demonstrates very similar flow rates and backpressure characteristics when the number of inlet valves in one kind of configuration is the same as that of outlet valves in another configuration. This indicates that the backflow from the pumping chamber to the inlet is basically the same as the backflow from the outlet to the pumping chamber. It is also found that an increase in the number of inlet valves or outlet valves can significantly suppress the backpressure fluctuation. The pump is very suitable for the conditions where a more accurate flow rate is required and wear and fatigue of check valves often occur. It will provide a new method for preventing the backflow in order to get accurate flow rates. We expect that this study will offer a better understanding of the time lag between diaphragm and valve movement. It is also expected that this work will provide new ideas about check-valve equipped piezoelectric membrane pumps.
